# The Relationship between Maternal Vitamin D Deficiency and Low Birth Weight Neonates 

**Published:** 2015-09

**Authors:** Nasrin Khalessi, Majid Kalani, Mehdi Araghi, Zahra Farahani

**Affiliations:** 1 Neonatal division, Ali Asghar Hospital, Iran University of Medical Sciences, Tehran, Iran; 2 Akbarabadi Hospital, Iran University of Medical Sciences, Tehran, Iran; 3 Maternal, Fetal and Neonatal Research Center, Tehran University of Medical Sciences, Tehran, Iran

**Keywords:** Infant, Low birth weight, vitamin D Deficiency

## Abstract

**Objective:** Maternal hypovitaminosis D may impair fetal growth and cause adverse pregnancy outcomes including intrauterine growth restriction and neonatal low birth weight. The aim of this study is to evaluate the relationship between maternal vitamin D status and neonate’s birth weight.

**Materials and methods:** A cross-sectional, descriptive analytical study was carried out in the nursery ward of 2 hospitals (Tehran-Iran) during one year (January 2011- January 2012). One hundred and two neonates were categorized into two groups, neonates with birth weight< 2500 gr (n=52) and neonates with birth weight>2500 gr (n = 50). Data regarding medical history, physical examination and anthropometric measurements of neonates were noted in a questionnaire. Birth time blood samples of their mothers were analyzed for serum 25-(OH)-vitamin D by ELISA method. Maternal vitamin D status was compared in two groups.

**Results:** Mean maternal vitamin D (vit D) level was 31.46 nmol/L. Forty eight percent of mothers had vitamin D deficiency, 27.5% had vit D insufficiency and 24.5% were normal. Mean maternal vitamin D level of LBW neonates was lower than other group; 25.05 vs. 38.13 (p = 0.001). All mothers of neonates with head circumference ≤ 33 cm also had vitamin D deficiency (p = 0.007).

**Conclusion:** Maternal Vitamin D deficiency may increase the risk of low birth weight neonate and modifying maternal nutrition behavior and their vit D level could be beneficial on pregnancy outcome.

## Introduction

Low birth weight (LBW) refers to term or preterm neonates with birth weight < 2500 gr. These neonates may be small for gestational age or have intrauterine growth restriction. Mortality rate in such neonates is 40 times more than those with normal weight (1). Some investigations highlighted the effect of micronutrients on birth weight (2). Vitamin D (vit D) has a key role in fetal growth by its interaction with parathyroid hormone and Ca2+ homeostasis. Studies confirmed that insufficient prenatal and postnatal levels of vit D have great effects on poor bone mineralization which have significant association with small for gestational age (SGA) births (3). SGA births are reported more frequent in pregnancies occurring in the winter with vit D deficiency (4). 

High Prevalence of vit D deficiency (about a billion) has been seen among people all over the world (2). Despite abundant and strong sunlight in Iran in most days of the year, vit D deficiency is a common problem among Iranian adult women due to their clothing style and lack of seasonal food. In this study, which was the first investigation in these medical centers (AliAsghar and Akbarabadi), we were intended to compare the maternal vitamin D status between LBW and normal birth weight neonates.

## Materials and methods

A cross-sectional, descriptive analytical study was carried out in the nursery ward of AliAsghar and Akbarabadi hospitals; 2 tertiary referral centers and also teaching hospitals affiliated to Iran University of Medical Sciences (Tehran-Iran) during a year (1 January 2011-1 January 2012). Neonatal physical examination and anthropometric assessments were done and recorded. The target population included term- normal birth weight (> 2500 gr), term neonates with birth weight < 2500 gr and their mothers (n = 107, p < 0.05, power = 80%). The exclusion criteria included mothers with preeclampsia, eclampsia, postpartum hemorrhage, insulin dependent diabetes mellitus, systemic and chronic disease, hematologic disorders, medication, twin pregnancy, drug abuse, and neonates with congenital malformation and infection (TORCH). A group of expert neonatologist (n = 5) designed a questionnaire based on review literatures, and then pretested it for 10 cases. Finally reliability and validity of the questionnaire have proved (Chronbakh α = 0.80). An expert nurse filled out questionnaires and recorded neonatal gender, birth weight and height, gestational age, mode of delivery, mother’s age, parity, race and ethnicity, mother’s education and clinical vit D deficiency symptoms. Immediately after birth, 5 ml of mother’s blood was collected, labeled and sent to laboratory to assay serum 25- (OH)-vit D level by 25-Hydroxy Vitamin D Enzyme immunoassay method (USA IDS Inc. P.O. Box 17063, Fountain Hills, AZ 85269-7063). Depending on mothers’ 25- (OH)-vit D level, all mothers were categorized in deficient (< 25 nmol/L), insufficient (25-50 nmol/L), normal (> 50nmol/L) and toxic level (> 250nmol/L) (2, 5).

The software package SPSS version 10 was used to perform the statistical analysis. Data are presented as mean + standard deviation for continuous variables and n% for categorical variables. The Chi Square, T test, ANOVA and Logistic regression were applied where applicable. The level of significant was considered p < 0.05.

The study was approved as a student thesis by the Medical Ethics Committee of IUMS (Iran University of Medical Science) (ID; 312) according Helsinki declaration. All participants gave informed consent before entering the study. Our gathered data were confidential and no extra cost was constrained on our participants.

## Results

Among 107 mothers, 5 mothers were excluded because of mothers' refusal, preeclampsia and insulin dependent diabetes mellitus.

Mean maternal serum vit D level in all 102 mothers with mean age 26.20 years and mean gestational age 38.73 weeks, was 31.46 nmol/L (4.3- 94 nmol/L). Some demographic characteristics are shown in table 1. 

**Table 1:** Demographic characteristics of participants

	**n = 102**
Gestational age (weeks)	37-42 (mean: 38.73)
Maternal age (years)	18-37 ( mean:26.20)
Mean birth weight (gr)	2985.33
Mean height (Cm)	49.75
Mean head circumference (Cm)	34.71
Ethnicity	Turk, Lor, Fars, Afghan

Of all mothers, 48% had vit D deficiency, 27.5% had vit D insufficiency and 24.5% were in normal range. 

Mean maternal serum vit D level in those with LBW newborns was significantly lower than in those with normal birth weight newborns (p=0.001). Maternal serum vit D status in both groups are demonstrated in table 2. 

**Table 2:** Maternal vit D serum level in LBW & normal birth weight neonates

	**Vit D serum level**	**Sufficiency** **n (%)**	**Insufficiency** **n (%)**	**Deficiency** **n (%)**
Mothers of LBW neonate	25.05 (SD = 20.16)	4 (7.7)	15 (30)	33 (62.3)
Mothers of normal birth weight neonate	38.13 (SD = 18.5)	21 (42 (	13 (26)	16 (32)

All 13 neonates with head circumference (HC)< 33cm were born to mothers with vit D deficiency in compare to 39 cases with HC > 33Cm, of which 20 were vit D deficient, 15 insufficient and 4 sufficient. Chi Square analysis signified the reverse relationship between maternal vit D deficiency and head circumference (p = 0.007).

Maternal vit D deficiency in neonates who were born by NVD, were lower than C/S delivered babies (p = 0.027).

There were no significant statistical differences in maternal vit D level between neonates with height ≤ 47 cm and neonates with height > 47 (p value = 0.054).

Mean maternal age was higher in vit D deficient mothers based on ANOVA Analysis (p = 0.031). However, based on Logistic regression test, maternal serum vit D deficiency caused neonatal LBW [p = 0.002, CI 95%:(-0.12)-(-0.003)] regardless of mothers’ age, and old age had no effect on both neonates’ birth weight (P = 0.44) or maternal serum vit D level (p = 0.8).

Regarding ethnicity, prevalence of vit D deficiency in Lor, Kord and Turk mothers was significantly higher than that in Fars mothers (p = 0.039).

There were no relationship between mother's parity or education and maternal vit D deficiency (p = 0.25, p = 0.43) respectively.

## Discussion

Vit D affects fetal growth by its interaction with Ca^2+^ homeostasis and parathyroid hormone. Few studies have been conducted to detect vitamin D status in mothers in Iran. In recent study, nearly half of our mothers had vit D deficiency. In another study which has performed in our country, vit D deficiency was found to have widespread incidence, especially in rural women (61.1%) in compare to the urban ones (46.2%) (6). Vit D deficiency is also reported to be very common in Pakistani mothers and infants (7). Hypovitaminosis D and osteomalacia in south Asian pregnant women have been widely reported, too (8). 

We found a significant correlation between LBW and maternal vit D deficiency. Our finding was compatible with study from Bodnar et al. They showed a positive relationship between vit D deficiency in white mothers and neonatal LBW, however; this finding was not seen in black mothers (9). Morley et al. have reported that among 354 newborns, the prevalence of LBW was higher in vit D deficient mothers (10). 

Safety of vit D supplements and fortification have been proved by some articles. Mothers using vitamin D supplementation (400-4000 IU/day) in 12-16 weeks showed lower preterm risks (11). It seems that vit D deficient mothers (25 Hydroxy Vit D < 30 ) have 2 fold more chance for giving birth SGA babies in compare to mothers with adequate vit D status (12). An investigation by the WHO has shown the role of micronutrients including vit D, in increasing about 77 gr birth weights and 25% decrease of LBW probability in Katmandu (2). 

Shin et al. have suggested that inadequate vit D intake during pregnancy is related to low neonatal birth weight and shorter infant height (11, 13), however we did not find any relationship between maternal vit D deficiency and neonatal height.

Low 25-(OH)-vit D level could be correlated with some mothers' diseases such as preterm labor, gestational diabetes mellitus and preeclampsia that affect on fetal growth (14-11), but these conditions were excluded from this study. Thus, the rate of maternal vit D deficiency may have been underestimated.

We saw that mothers with vit D deficiency gave birth neonates with head circumference (HC) <33cm. In consistent to our results, Aghajafari et al. indicated a significant association between small for gestational age infants (with small fetal growth indices) and mother's vit D insufficiency (15). On the other hand Hashemipour et al. showed an independent correlation between neonatal head circumference with maternal vitamin D level (16).

Although a significant difference was found between the mode of delivery and mother’s vit D status, our finding was against investigationfrom Dror et al. (17).

No relationship was found between mother’s education and the level of vit D, unlike the Scholl et al. study, in which reluctance to use prenatal multivitamins and other dietary supplements was reported among illiterate women (18).

In addition, some studies emphasize that the ethnicity and race both have a role in vit D deficiency in women and neonates (19). In our study, prevalence of vit D deficiency in Lor, Kord and Turk mothers was significantly higher than Fars mothers (p=.039). Therefore, it seems that ethnicity could be a crucial factor in this regard.

Although our study did not evaluate the effects of maternal nutrition, supplemental intake and the amount of sunlight exposure on the vit D status, we speculate that these factors should be considered in future studies.

## Conclusion

In conclusion as high prevalence of vit D deficiency has been seen among women especially in developing countries, this study shows that neonatal low birth weight (LBW) could be related to maternal vitamin D deficiency. Modifying maternal nutrition behavior and vit D level could be beneficial on prevention of low birth weight, however more research in this field seems contribute to an improvement in maternal and neonatal health.
